# Estimation of the Number of Passengers in a Bus Using Deep Learning

**DOI:** 10.3390/s20082178

**Published:** 2020-04-12

**Authors:** Ya-Wen Hsu, Yen-Wei Chen, Jau-Woei Perng

**Affiliations:** Department of Mechanical and Electro-Mechanical Engineering, National Sun Yat-sen University, Kaohsiung 804201, Taiwan; ste121230@hotmail.com (Y.-W.H.); wmit34522@gmail.com (Y.-W.C.)

**Keywords:** crowd density estimation, deep learning, object detection, passenger counting, YOLOv3

## Abstract

For the development of intelligent transportation systems, if real-time information on the number of people on buses can be obtained, it will not only help transport operators to schedule buses but also improve the convenience for passengers to schedule their travel times accordingly. This study proposes a method for estimating the number of passengers on a bus. The method is based on deep learning to estimate passenger occupancy in different scenarios. Two deep learning methods are used to accomplish this: the first is a convolutional autoencoder, mainly used to extract features from crowds of passengers and to determine the number of people in a crowd; the second is the you only look once version 3 architecture, mainly for detecting the area in which head features are clearer on a bus. The results obtained by the two methods are summed to calculate the current passenger occupancy rate of the bus. To demonstrate the algorithmic performance, experiments for estimating the number of passengers at different bus times and bus stops were performed. The results indicate that the proposed system performs better than some existing methods.

## 1. Introduction

In recent years, with the rapid development of technologies such as sensing, communication, and management, improving the efficiency of traditional transportation systems through advanced technological applications is becoming more feasible. Therefore, intelligent transportation systems have gradually become a focus of transportation development around the world. Currently, many related applications exist in the field of bus information services; for example, people can utilize a dynamic information web page or a mobile app to inquire about the location and arrival time of every bus. If more comprehensive information is provided on existing bus information platforms, the quality of public transport services will be significantly improved. Thus, the number of passengers willing to use public transport will increase. The information regarding the load on each bus is critical to the control and management of public transport. The ride information includes the number of passengers entering and leaving at each stop and the number of passengers remaining on the bus. Through intelligent traffic monitoring, passengers can preview a bus’s occupancy status in real time and then make a decision based on the additional information and evaluate the waiting time. Furthermore, passenger transport operators can manage vehicle scheduling based on this information; thus, operational costs depending on whether service quality is degraded or not are reduced effectively while providing passengers with more useful ride information.

In the past, some research groups have explored the counting of passengers on buses. Luo et al. proposed extracting footprint data through contact pedals to determine the directions in which passengers entered and left the bus [1]. Torriti and Landau proposed radio-frequency identification technology to implement passenger counting, although the recognition result is susceptible to the position of the RF antenna and the direction of radiation [2]. With the popularity of surveillance cameras and advances in computer vision technology, image-based people-counting methods have been continuously proposed [3,4,5,6,7,8,9,10]. References [3,4,5,10] employed images from actual buses as experimental scenes. The images were captured from a camera mounted on the ceiling of a bus, with an almost vertical angle of view. In these works, the heads of the passengers were a noticeable detection feature in the images, and their methods included motion vectors, feature-point-tracking, and hair color detection. The authors in a recent article [10] proposed a deep learning-based convolutional neural network (CNN) architecture to detect passengers’ heads. Reference [11] proposed a counting method by combining the Adaboost algorithm with a CNN for head detection. This study was divided into three phases; namely, two offline training phases and one online detection phase. The first phase used the Adaboost algorithm to learn the features obtained from the histogram of oriented gradients (HOGs). The second phase established a new dataset from the preliminary results detected in the first phase as the modeling data for training the CNN. The resulting model was used as a classifier for the head feature in the online detection phase.

Based on the results of the above references, although object detection technology development is mature, in a complex environment and crowd-intensive situation, achieving high detection performance with a single algorithm is improbable. Existing works related to the number of passengers on a bus are generally concerned with the image of the bus door and ignore the overall situation inside the bus. However, as time flows, the accuracy of the number of passengers on the bus significantly drops owing to the accumulation of the counting errors of passengers getting on and off of the bus. Only a few studies have directly calculated numbers of passengers on buses. Reference [12] proposed a shallow CNN architecture suitable for estimating the level of crowdedness in buses, which was a modification of the multi-column CNN architecture. A fully connected layer was added to the network output, and then the output of the model was classified into five levels of crowdedness [12].

In terms of crowd density estimation, the regression model was used in [13,14,15,16,17,18] to calculate the number of people in a crowd of a public scene. This method solves the occlusion problem between the people in a crowd. Hence, the primary step is to obtain the necessary characteristic information from the image, such as edge, gradient, and foreground features. Then, linear regression, ridge regression, or Gaussian process regression is used to learn the relationship between the crowd features and the number of people. In [19], the image is first divided into small blocks one by one, and multiple information sources are used to overcome the problem of overly dense and complex background environments. The number of individuals present in an extremely dense population is calculated from a single image. At various scales, the HOG detection method and Fourier analysis are used to detect the heads of people. The points of interest are used to calculate the local neighbors. Then, each block establishes the number of people from these three different methods. Finally, the total number of people in the image is the sum of the number of people in each block. The author in [20] proposed a new supervised learning architecture that uses the crowd density image corresponding to an original crowd image to obtain the number of people in an area through regional integration. The practical loss function approach is defined as the difference between the estimated density map and the ground truth density map, and is used to enhance the learning ability. This procedure helps provide the correct direction for deep learning applications in crowd density estimation.

The CNN architecture is used in [21,22,23,24,25] to solve the problems of severe occlusion and distortion of the scene perspective. The CNN implements an end-to-end training process that inputs the raw data and outputs the final result without the need to pass through foreground segmentation or feature extraction. Using supervised learning, the original image through multiple convolutional layers can produce the corresponding density map. The authors in [26,27] proposed the use of a multi-column CNN (MCNN) architecture to map the relationship between crowd images and density images. The filters corresponding to the different columns of the convolutional layers can perform operations on images of different input sizes. Thus, the problem concerning the different sizes of peoples’ heads in an image is solved. Finally, the three-column CNN is linearly weighted to obtain the corresponding number of people in the original image. The author in [28] reported that crowd estimation involves three characteristics; namely, the entire body, head, and background. The body feature information can help with judging whether a pedestrian exists in a specific position. However, the existing density labeling method is mostly marked according to the method mentioned in [20], so the semantic structure information is added to improve the accuracy of the data labeling, and the estimation of the number of crowd scenes can be more effective. The authors in [29] improved the scale-adaptive CNN (SaCNN) proposed in [25]. Adaptive Gaussian kernels are used to estimate the parameter settings for different head sizes. The original image is reduced to a size of 256×256, which improves the diversity of training samples and increases the network’s generalization ability. The authors in [30] proposed a multi-column multi-task CNN (MMCNN), which defines the input image as a single channel of 960×960 when training the network model image. This image is evenly divided into 16 non-overlapping blocks during the training phase to avoid network over-fitting and low generalization during the process. To solve the problem in the evaluation phase of the model, the image is divided into 120×120 up to 480×480 different sizes for input; then, all the block images are sequentially extracted, and the output of the three columns is merged to output the multi-tasking result. The outputs are a mask of the crowd density map, congestion degree analysis, and masks of the foreground and background.

In [12], although a bus congestion evaluation is involved, the result of the network output is simply at the classification level. In the present study, we propose the use of a deep learning object detection method and the establishment of a convolutional autoencoder (CAE) to extract the characteristics of passengers in crowded areas to evaluate the number of people on a bus. The results of these two methods are summed into the total number of passengers on the bus.

The rest of the paper is organized as follows. In Section 2, the proposed system is described. In Section 3, the proposed methodology is introduced. Section 4 presents the experimental results and evaluates the proposed schemes. Finally, conclusions and discussions on ideas for future work are provided in Section 5.

## 2. Overview of the Proposed System

### 2.1. Systems Architecture

**Figure 1:**
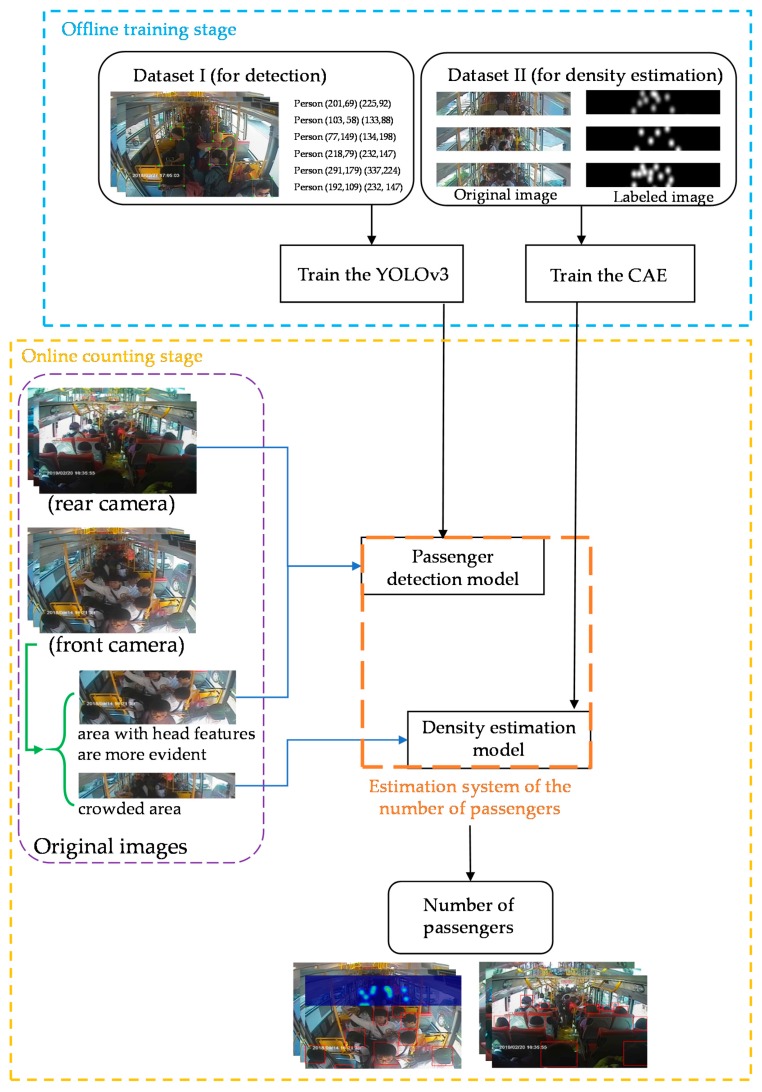
Flowchart for estimation of the number of passengers on a bus.

The proposed system estimates the number of passengers on a bus based on deep learning. The process of the developed system is shown in Figure 1. The system first pre-trains two deep learning network models in an offline manner. Here, the main difference is that the area in which the head features of a passenger are visible in an image picture is established by employing object detection, while the area where space is prone to crowding and head features are not apparent is based on the density method to estimate the number of passengers. At the stage of the online passenger number estimation, the images to be tested are divided into crowded areas and areas with more evident head features, which are then input into the trained density estimation model and passenger detection model, respectively. The system can instantly output the number of people on the bus. The two counting sections of the system are briefly described as follows:

1. Passenger counting based on object detection: For areas where the head features of passengers are more visible, the deep learning object detection method is employed to calculate the number of passengers in the image. The you only look once version 3 (YOLOv3) network model with a high detection rate was selected.
2. Passenger counting based on density estimation: We propose a CAE architecture suitable for scenarios of crowded areas on a bus. This model filters all objects in the original image that do not possess passenger characteristics and outputs the information characteristics of the passengers in the image.

### 2.2. Systems Database

**Figure 2:**
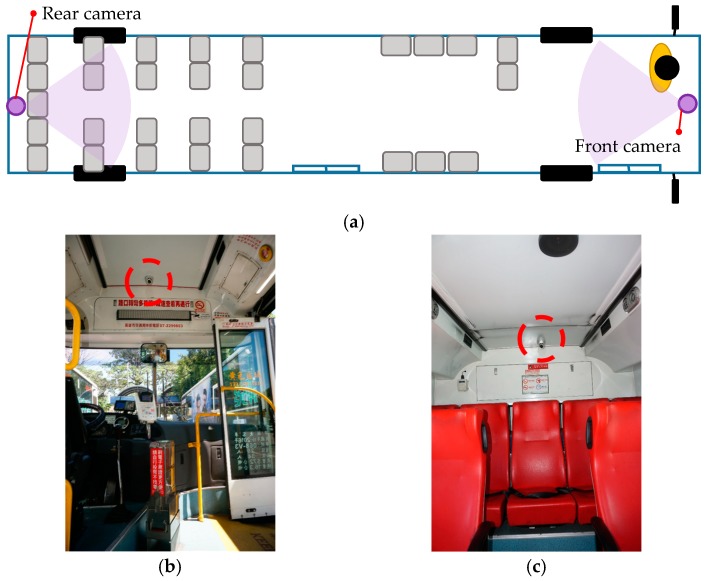
Positions of the surveillance cameras: (**a**) internal configuration of the bus—images of (**b**) front camera and (**c**) rear camera.

All the videos used to produce the samples were obtained from the monitoring system of a city bus in Kaohsiung, Taiwan. We utilized these image data to create a proprietary database of passengers for training the proposed neural network model. The surveillance cameras were mounted inside the front and rear of the bus at the height of approximately 240 cm from the bus floor, as shown in Figure 2a. The actual positions are shown in Figure 2b,c.

**Figure 3:**
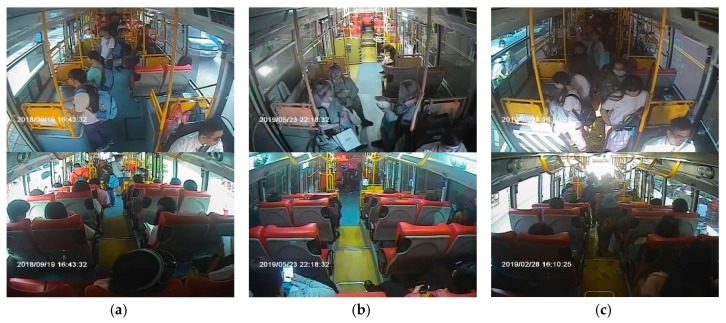
Examples of in-vehicle images obtained from each surveillance camera: (**a**) daytime scene, (**b**) nighttime interior lighting scene, (**c**) scenes affected by light.

For passenger counting based on object detection, a total of 5365 images from inside the bus recorded from February to November 2018 were utilized as the training set. Moreover, 500 sets of in-vehicle images from November 2018 to May 2019 were utilized for the experimental tests. We defined the images simultaneously captured from the front and rear cameras as the same set. In this dataset, the periods of different lighting conditions are included, which are the daytime scene, nighttime interior lighting scene, and scenes affected by light, as shown in Figure 3a–c. Some of the most common situations in the images of a bus are shown in Figure 3; the pictures in the first and second rows present different cases for the front and rear cameras, respectively. In the image of the daytime scene, we can easily observe the current number of passengers on the bus. Meanwhile, in the nighttime scene, images from the front camera are sometimes disturbed by the fluorescent light of the bus, and images from the rear camera show the colors of some passengers’ heads as similar to the scene outside the bus. In the scenes affected by light, images from the front camera have uneven light and shade due to sunlight exposure.

**Figure 4:**
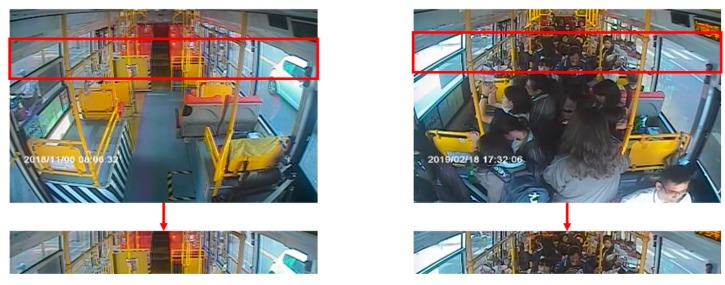
Crowded area definition.

In the images from the front camera, the range of passenger congestion occurs in a fixed area. Therefore, we define the space of passenger congestion on the bus, as shown in Figure 4. When this area is filled with passengers, serious occlusion problems generally occur, resulting in less distinct head characteristics of passengers. A convolution auto-encode model based on a density map was used to predict the number of passengers in this area.

**Figure 5:**
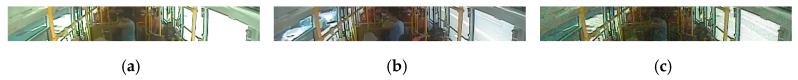
Data augmentation: (**a**) original image, (**b**) darkened, and (**c**) added noise.

For training the passenger density data, a total of 3776 images of crowded areas inside the bus were marked. To increase the diversity of training images, data augmentation was performed on each image by Gaussian noise, and the brightness was adjusted, as shown in Figure 5. The original dataset was augmented to 11,328. The images of crowded areas were divided into training and validation samples to train the neural network, and the segmentation ratio of the two samples was 7:3.

## 3. Methodology

### 3.1. Passenger Counting Based on Object Detection

YOLO is an end-to-end CNN often used for object detection and recognition and has been successfully applied to many detection fields, such as traffic sign, pedestrian, and vehicle detection. By developing the detection process into a single deep neural network, the predicted bounding box and classification probability can be obtained simultaneously to achieve faster and higher-precision object detection. New versions of the YOLO model have been released, and its detection performance continues to improve. Owing to its high efficiency, we directly used the YOLOv3 algorithm [31] based on the Darknet-53 architecture as our detection model. The process of the YOLO structure for passenger detection is described below.

First, images with ground-truth bounding boxes of heads and the corresponding classification labels are input to the network. The input images are reduced to 448×448 resolution and divided into S×S grids. If the object’s center falls into the grid, each grid is responsible for predicting B bounding boxes of objects. Each bounding box contains five information factors; namely, $P_{x},\;P_{y},\;P_{w},\;P_{h},\;c$, where $P_{x}$ and $P_{y}$ represent the center coordinates of the bounding box relative to the bounds of the grid cell. $P_{w}$ and $P_{h}$ are the width and height predicted relative to the entire image, respectively. The confidence c is defined using Equation (1).

(1) $$c=P_{0}\times P_{IOU}.$$

Here, $P_{0}$ represents the probability of the box containing a detection object and $P_{IOU}$ is the intersection over union between the detection object and the predicted bounding box.

Each bounding box corresponds to a degree of confidence. If there is no target in the grid cell, the confidence is 0. If there is a target, the confidence is equal to $P_{IOU}$.

YOLO’s loss function $\lambda_{loss}$ is calculated using Equation (2).

(2) $$\lambda_{loss}=\sum_{i=0}^{S^{2}}E_{coord}+E_{IOU}+E_{class}.$$

Here, $E_{coord}$ represents the coordinate error, $E_{IOU}$ is the $c=P_{0}\times P_{IOU}$ error, and $E_{class}$ is the classification error between the predicted results and the ground truth.

As the number of training iterations increases, the weight parameters of the network model are continuously updated until the loss function reduces to less than a preset value, and the network model is considered to be completely trained.

### 3.2. Passenger Counting Based on Density Estimation

#### 3.2.1. Inverse *K*-Nearest Neighbor Map Labeling

**Figure 6:**
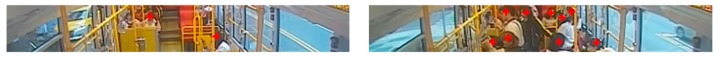
Labelled head positions.

First, the position of each passenger’s head in the crowd image is labeled, as shown in Figure 6. The red cross symbol in the figure represents the head coordinates of a person to be marked. If there is a head at pixel $\left(x_{h},y_{h}\right)$, it is represented as a delta function $\delta (x-x_{h},y-y_{h})$. An image with N heads labeled is depicted by Equation (3).

(3) $$H(x)=\sum_{h=1}^{N}\delta (x-x_{h},y-y_{h}).$$

To convert the labeled image into a continuous density map, a common method involves performing a convolution operation on H(x) and a Gaussian function $G_{\sigma }(x)$ to obtain the density map $D_{g}$, as shown by Equation (4).

(4) $$D_{g}(x,f(\cdot ))=H(x)*G_{\sigma }(x)=\sum_{h=1}^{N}\frac{1}{\sqrt{2\pi }f(\sigma_{h})}\exp\left(-\frac{{\left(x-x_{h}\right)}^{2}+{\left(y-y_{h}\right)}^{2}}{2f{\left(\sigma_{h}\right)}^{2}}\right),$$

where $\sigma_{h}$ is a size determined by the k-nearest neighbor (*k*NN) distance of each head position $\left(x_{h},y_{h}\right)$ from other head positions (a fixed size is also used), and *f* is a manually determined function for scaling $\sigma_{h}$ to decide the kernel size of the Gaussian function.

We adopt inverse *k*NN (i*k*NN) maps as an alternative labeling method from the commonly used density map. According to [32], the i*k*NN graph performs better than density maps when there are ideally selected spread parameters. Here, f is defined as a simple scalar function $f(\sigma_{h})=\beta \sigma_{h}$, and β is a scalar that is manually adjusted. The full *k*NN map is defined by Equation (5).

(5) $$K(x,k)=\frac{1}{k}\sum \underset{k}{\min}\left(\sqrt{{\left(x-x_{h}\right)}^{2}+{\left(y-y_{h}\right)}^{2}},\forall h\in H\right),$$

where H is the list of all head positions. The above formula calculates the *k*NN distance from each pixel (*x*, *y*) to each head position $\left(x_{h},y_{h}\right)$.

**Figure 7:**
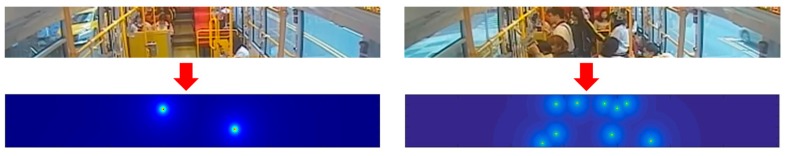
Inverse *k*NN map of the crowded area.

The calculation to generate the i*k*NN map is depicted as Equation (6). The i*k*NN map is shown in Figure 7.

(6) $$M=\frac{1}{K(x,k)+1}$$

#### 3.2.2. Architecture of the Proposed Convolutional Autoencoder

Masci et al. proposed a deep neural network architecture CAE in 2011 [33]. A CAE combines the traditional autoencoder of unsupervised learning with the convolutional and pooling operations of CNNs. Two implementation processes are used: encoding and decoding. The model is trained by comparing the encoded and the original data, so the decoded data can be restored to the original data as much as possible.

**Figure 8:**
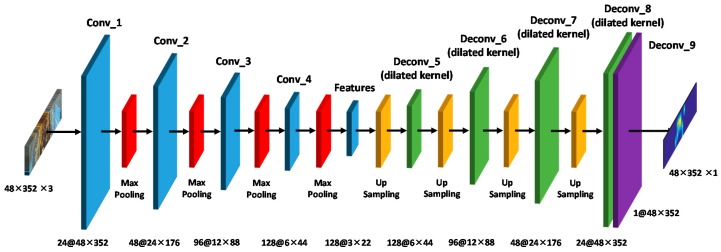
Architecture of the proposed convolutional autoencoder (CAE).

The head feature extraction in crowded areas in this study was mainly based on the CAE network. In the offline stage, the color image containing a passenger’s head and the i*k*NN map were first input to train the CAE model. Then, the image passed the trained model to obtain a pure image of the head feature that filtered the other objects. The number of passengers in the crowded area in the image was then calculated by integration. Figure 8 shows the CAE architecture applied in this study. In the network architecture, 9 convolutional layers were used, and the input was a color image with a size of 48 × 352 in the crowded area we previously defined. Excluding the last convolutional layer, batch normalization was used after each convolutional layer to prevent the neural network from overfitting. Further, a rectified linear unit was used as an activation function to introduce nonlinear factors that overcome the gradient disappearance. The first to forth convolutional layers each use a max-pooling with the kernel size of 2 × 2 to reduce the image, while the fifth to eighth convolutional layers use up-sampling with the kernel size of 2 × 2 to enlarge the image. The first convolution layer has 24 filters, each of size 9 × 9; the second has 48 filters, each of size 7 × 7; the third has 96 filters, each of size 5 × 5; and the fourth has 128 filters, each of size 3 × 3. Inspired by [34], to enlarge receptive fields and extract deeper features without losing resolution, the dilated kernels with the dilation rate of 2 were used for the back-end. The first deconvolution layer has 128 filters, each of size 3 × 3; the second has 96 filters, each of size 5 × 5.; the third has 48 filters, each of size 7 × 7; and the fourth has 24 filters, each of size 9 × 9. Because the final output image is a single channel, only one filter was used, and the sigmoid function was used as an activation function. To make the size of the output data consistent with the size of the input data and avoid loss of image space information in the output density map, up-sampling was used in the convolutional layer after the fifth layer to restore the original image size.

## 4. Experimental Results and Discussion

### 4.1. Introduction to the Experimental Scene

The 100th route traveled by a bus in Kaohsiung was utilized as the research scenario as it is an important transportation route in Kaohsiung City. This bus route passes multiple department stores and schools; hence, many passengers are students travelling to and from class, making the bus particularly crowded.

**Figure 9:**
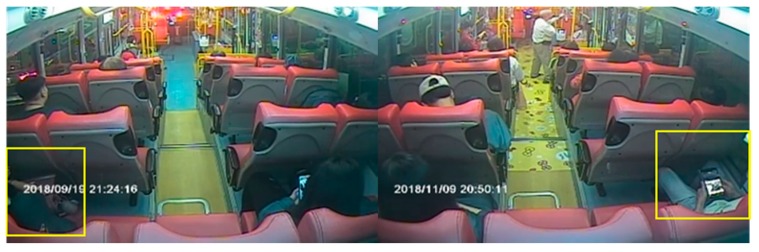
Occlusion range of the rear camera.

Before evaluating the performance of each detection model, we first define the calculation method of the image of a passenger on the bus. In the image of the rear camera in the bus, there are a total of 21 passenger seats. Images of passengers on the left and right sides of the last row are susceptible to the positioning of the lens. As a result, only the lower body is often exposed in the image, as shown in Figure 9. In the training of the deep learning model, we use the head of the passenger as the main training feature, so information of these two seats in the rear camera view is ignored.

**Figure 10:**
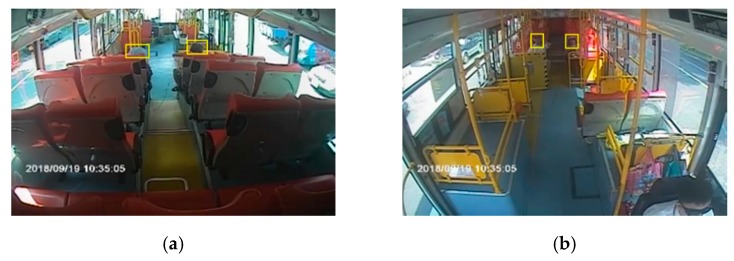
Seating area definition: (**a**) rear camera; (**b**) front camera.

Additionally, in the rear camera view, passengers alongside the aisle of the first row in the rear seating area may be blocked by passengers in the second row of seats, as shown in Figure 10a. In the front camera view, the passengers seated in the two seats in the middle of the first row are clearly seen compared to the rear camera view, so this part of the passenger information was considered within the estimation range of the front camera, as shown in Figure 10b.

Therefore, according to the aforementioned calculation methods of the front and rear cameras, we counted the actual number of people in the 500 sets of bus test data manually, and the statistics of this dataset are the model testing data to be used.

### 4.2. Calculation of the Total Number of Passengers

**Figure 11:**
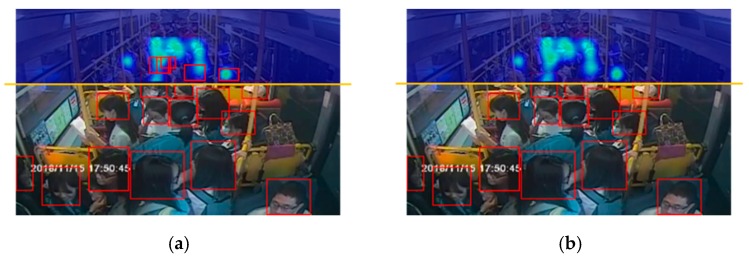
Repeat detection area: (**a**) without correction and (**b**) with correction.

In the front camera view, passengers in the edge of the crowded area might be recounted by the CAE and YOLOv3. Therefore, to obtain more accurate estimation results, we established a dividing line on the image to deal with this problem. As shown in Figure 11a, in the overlapping detection area, we determined the position of the center point of the object bounding box. When the center point of the object is within the defined density estimation area, this detection object belongs to the counting result of the density estimation. By segmenting the detection area and the density estimation area through the dividing line, the problem of repeated counting can be avoided, as shown in Figure 11b.

In the rear camera view, there is no occlusion problem. Therefore, we mainly used the YOLO detection method to count the number of passengers in the seats and aisles of the bus. Finally, the results of the CAE density estimation and YOLO detection can be summed to obtain the current number of passengers on the bus.

### 4.3. Evaluation of Passenger Number Estimation

After defining the test dataset, to verify the effectiveness of the proposed algorithm in passenger number estimation, we compare it to two methods: SaCNN [25] and MCNN [26]. To account for the performances of the different detection models, we used the mean absolute error (MAE) and root mean squared error (RMSE), as shown in Equations (7) and (8), to evaluate the effectiveness of models based on the references [28,29,30], respectively. $N_{img}$ is the number of test images, $x_{g}^{i}$ is the actual number of passengers in the test image, and ${\hat{x}}_{p}^{i}$ is the number of passengers on the bus estimated by the different methods. Table 1 shows the performances of the different methods for 500 sets of test data from the bus images.

(7) $$\mathrm{MAE}=\frac{1}{N_{img}}\sum_{i=1}^{N_{img}}\left|x_{g}^{i}-{\hat{x}}_{p}^{i}\right|,$$

(8) $$R\mathrm{MSE}=\sqrt{\frac{1}{N_{img}}\sum_{i=1}^{N_{img}}{\left(x_{g}^{i}-{\hat{x}}_{p}^{i}\right)}^{2}}.$$

**Figure 12:**
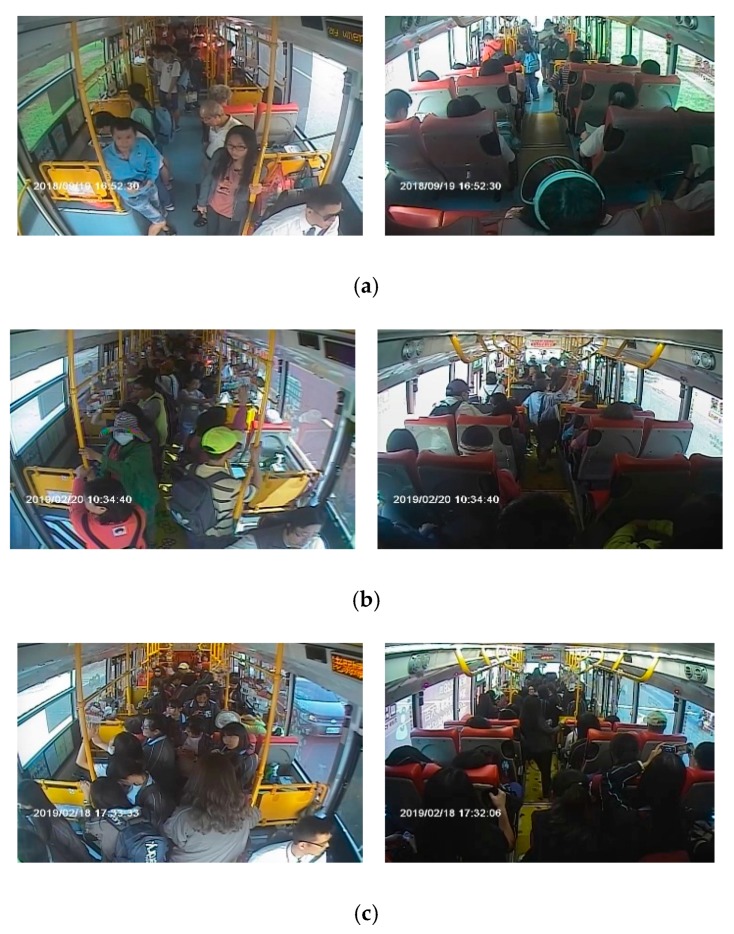

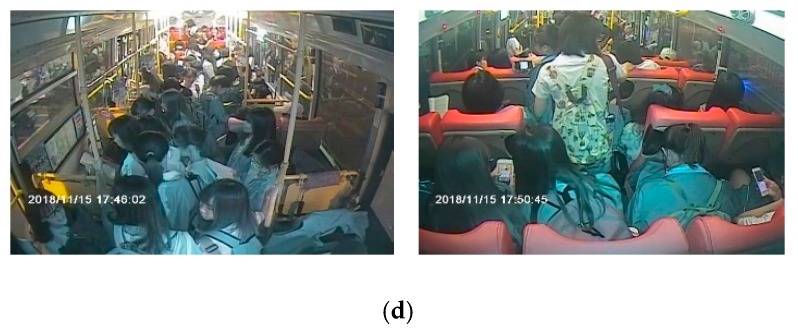
Examples of crowded scenes: (**a**) 25 passengers, (**b**) 30 passengers, (**c**) 35 passengers, and (**d**) 40 passengers.

The estimation performance results shown in Table 1 indicate that if only a single neural network architecture was used to estimate the number of passengers in the bus scenario, whether it is a density-based method or a detection-based method, there would be a significant error in the model performance evaluation. In our experimental scene, the image field of view is relatively small, resulting in extreme differences in the size of the passengers’ heads, as seen closer and farther in the image. As a result, estimates using a single network architecture are poor. To further investigate the performance of the proposed algorithm, we established in the original bus passenger dataset that when the bus interior contains more than 25 passengers, it is considered to be more crowded, as shown in Figure 12, wherein 104 sets of images belong to this category. In the next part, only the YOLOv3 detection method is analyzed and compared with the proposed method in this study.

Figure 12a shows a picture of 25 passengers on the bus. Although there is space in the aisle section, as seen in the front camera view, the seating area seen in the rear camera is almost full. Figure 12b shows 30 passengers inside the bus. The front camera shows the passengers on both sides of the aisle, and there are many passengers in the defined crowded area of the image. Figure 12c shows 35 passengers in the bus. This picture shows that most of the space, as seen in the front camera, has been filled, and the seating area seen in the rear camera is full. Figure 12d shows 40 passengers in the bus. The space shown in the front camera view is full, and there are a few passengers standing in the area near the door. Few passengers can be seen standing on the aisle in the rear camera image as well. Based on this, the crowded passenger dataset was explicitly selected for further analysis and comparison. The analysis results are shown in Table 2. The estimation results indicate that the performance of the proposed algorithm is better than when using only the YOLOv3 detection method. From the estimation results, we observed that the most significant performance difference between the two methods occurred in the defined crowded area.

**Figure 13:**
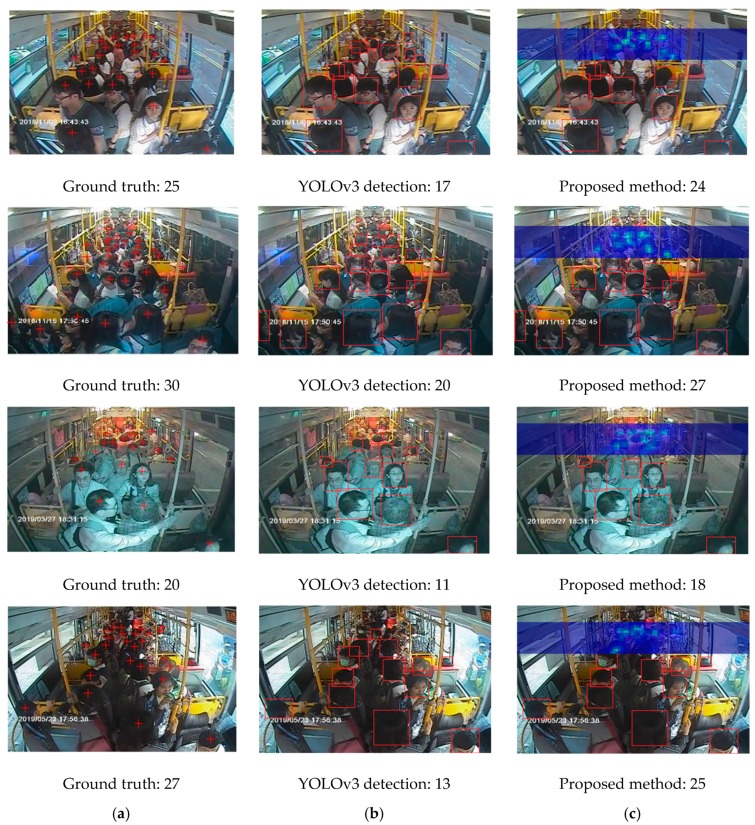
Estimation results for each method: (**a**) original image, (**b**) YOLOv3 only, and (**c**) proposed architecture.

Figure 13a–c presents the front camera images from the bus, and the detection results from employing YOLOv3 and from the proposed architecture of this study, respectively. Take the first row of images as an example. The ground truth of the number of passengers in the image is 25. The results of the YOLOv3 detection and the proposed method are 17 and 24, respectively. The result of YOLOv3 detection, in Figure 13b, proves that more detection failures occur in the defined crowded passenger areas only if this single detection algorithm is employed to estimate the congestion of passengers for the front camera. As shown in Figure 13c, the density estimation network method can compensate for the low detection rate in the crowded area, and the density image presents the distribution of each passenger in the crowded area, so the result of the final estimation of passengers in the bus is closer to the actual number of passengers in the bus, compared with that from a single detection method.

### 4.4. Evaluation of System Performance in Continuous Time

To assess the effectiveness of the proposed model, we also performed model tests on continuous time bus images. The selected scenes can be divided into three main conditions: afternoon, evening, and nighttime. The reason for choosing these conditions is that in the afternoon, the image inside the bus is more susceptible to light exposure, while in the evening, many crowded situations occur. At night, the image of the bus is affected by the interior fluorescent lights and scenes outside the bus. In the following section, we introduce these three continuous time scenes individually and evaluate the estimation results of the passengers inside the vehicle.

#### 4.4.1. Estimated Results (Afternoon)

**Figure 14:**
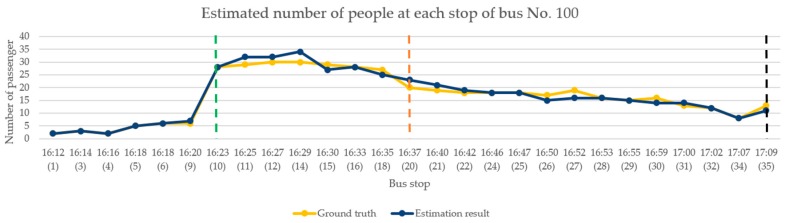
Continuous image analysis for afternoon.

In the performance analysis for afternoon time, we selected the test video from 4:00–5:00 p.m. on 23 March 2019. This period was selected because of the relationship between the driving route and the direction of the bus. During this period, the sunlight caused uneven brightness in the image of inside the bus. Moreover, there were students travelling from school during this period, so the congestion of passengers can also be observed. The occurrence of the above situations can verify the effectiveness of the proposed model framework. Figure 14 shows the change in the number of passengers when the bus arrives at each bus stop. The bus stop numbers are arranged chronologically from left to right on the horizontal axis. During this period, the bus had a total of 27 stops. The yellow polyline shown in the figure indicates the actual number of passengers on the bus. The dark blue polyline is the number of passengers estimated by the proposed framework. The green dotted line represents a large number of passengers entering the bus at that stop, and the number of people on the bus increased to approximately 30. The orange dotted line represents a large number of passengers leaving the bus at that stop, where approximately half the passengers got off. The black dotted line is the bus terminal.

According to Figure 14, although there are errors in the estimation of the number of people at a few stops when the image was affected by lighting, the overall analysis results are consistent with the current number of passenger changes on the bus. Table 3 shows the results of the model performance evaluation of the continuous images of the bus in the afternoon. Five consecutive seconds of the image were taken for analysis after passengers get on the bus, where each second is represented by one frame. Finally, the median number of passengers estimated by the five consecutive images was considered as the result of this stop.

#### 4.4.2. Estimated Results (Evening)

**Figure 15:**
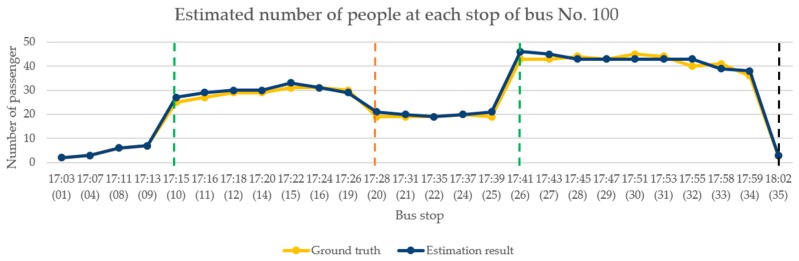
Continuous image analysis for the evening.

For the analysis of the performance of the model in the evening, we selected the test recording for the period from 5:00–6:00 p.m. on 23 May 2019. As shown Figure 15, the bus made 26 stops during this period. This period was chosen because the bus passes three schools along its route and students leave school around this time. As students started leaving school for the day, passenger congestion started increasing on the bus. Therefore, the evaluation performance of the proposed model architecture was more extensively verified. The green dotted line indicates that a large number of passengers entered the bus at that stop; the number of passengers on the bus at the stop of the first green line increased to approximately 30, and the number at the stop of the second green line increased to approximately 45. The orange dotted line represents the large number of passengers who left the bus at that stop—approximately half the passengers got off the bus. The black dotted line represents the bus terminal. Table 4 shows the results of the performance evaluation of the continuous images of the bus in the evening.

#### 4.4.3. Estimated Results (Nighttime)

**Figure 16:**
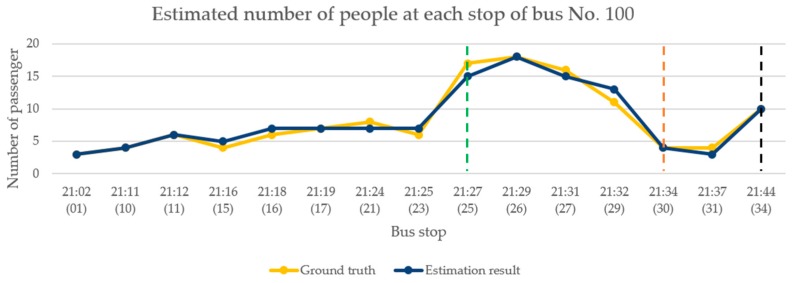
Continuous image analysis for nighttime.

In the performance analysis of the model at night, the selected test video record was from 9:00–10:00 p.m. on 23 May 2019. This period was chosen because the brightness of the bus image was affected by interior lighting and the outside scenery. Figure 16 presents the change in the number of passengers when the bus arrived at each bus stop. During this period, the bus made a total of 16 stops. The yellow polyline shown in the figure indicates the actual number of passengers on the bus. The dark blue polyline indicates the number of passengers estimated by the proposed framework. The green dotted line indicates a large number of passengers boarding the bus at that stop; the orange dotted line indicates a large number of passengers leaving the bus at that stop; and the black dotted line indicates the bus terminal. Table 5 presents the results of the performance evaluations of continuous image modeling of the bus at nighttime.

## 5. Conclusions

In the existing bus passenger-counting literature, cameras were installed above the doors to calculate the numbers of passengers getting on/off buses. However, research on directly estimating the number of passengers on a bus has not been explored. In this study, front and rear cameras placed on the bus are used to estimate the number of passengers. The algorithm used is a combination of a deep learning object detection method and the CAE architecture. The CAE density estimation model was used to extract the passenger features of the crowded area, and YOLOv3 was used to detect the areas with more apparent head features. Then, the results obtained by the two methods were summed to estimate the number of passengers in the vehicle. Moreover, this result was compared with other methods. In the final performance evaluation, the MAEs for the bus passenger dataset and the crowded dataset were 1.35 and 1.98, respectively. In these experiments, the RMSEs were 2.02 and 2.66, respectively. Furthermore, we estimated the number of passengers on a bus for three consecutive times; namely, afternoon, evening, and nighttime. The results were consistent with the variations in passenger numbers at each stop.

Although the algorithm used in this study has better estimation performance, the proposed CAE density estimation network model is still susceptible to light exposure, which reduces accuracy. This issue will be addressed in our future work. In the future, we also hope to combine the proposed algorithm for estimating the number of passengers with the method of counting passengers getting on and off a bus to provide more reliable information in terms of bus load.

## Figures and Tables

**Table 1:** Performances of different methods for the bus passenger dataset.

Bus Passenger Data Set (500 sets)		
Method	MAE	RMSE
SaCNN [25]	3.25	4.37
MCNN [26]	2.96	3.85
Density-CAE	2.31	2.98
YOLOv3	2.54	3.17
Density-CAE + YOLOv3	1.35	2.02

**Table 2:** Performances of different methods for the crowded bus dataset.

Crowded Dataset (104 Sets)		
Method	MAE	RMSE
YOLOv3	4.93	5.31
Density-CAE + YOLOv3	1.98	2.66

**Table 3:** Evaluation of the continuous image model for afternoon.

	MAE	RMSE
Density-CAE + YOLOv3	1.11	1.66

**Table 4:** Evaluation of continuous image model for the evening.

	MAE	RMSE
Density-CAE + YOLOv3	1.15	1.52

**Table 5:** Evaluation of continuous image model for nighttime.

	MAE	RMSE
Density-CAE + YOLOv3	0.63	0.94

## References

[1] Luo Y., Tan J., Tian X., Xiang H. A device for counting the passenger flow is introduced. Proceedings of the IEEE International Conference on Vehicular Electronics and Safety.

[2] Oberli C., Torriti M.T., Landau D. (2010). Performance evaluation of UHF RFID technologies for real-time passenger recognition in intelligent public transportation systems. IEEE Trans. Intell. Transp. Syst..

[3] Chen C.H., Chang Y.C., Chen T.Y., Wang D.J. People counting system for getting in/out of a bus based on video processing. Proceedings of the International Conference on Intelligent Systems Design and Applications.

[4] Yang T., Zhang Y., Shao D., Li Y. (2010). Clustering method for counting passengers getting in a bus with single camera. Opt. Eng..

[5] Chen J., Wen Q., Zhuo C., Mete M. Automatic head detection for passenger flow analysis in bus surveillance videos. Proceedings of the IEEE International Conference on Vehicular Electronics and Safety.

[6] Hu B., Xiong G., Li Y., Chen Z., Zhou W., Wang X., Wang Q. Research on passenger flow counting based on embedded system. Proceedings of the International IEEE Conference on Intelligent Transportation Systems (ITSC).

[7] Mukherjee S., Saha B., Jamal I., Leclerc R., Ray N. A novel framework for automatic passenger counting. Proceedings of the IEEE International Conference on Image Processing.

[8] Xu H., Lv P., Meng L. A people counting system based on head-shoulder detection and tracking in surveillance video. Proceedings of the International Conference On Computer Design and Applications.

[9] Zeng C., Ma H. Robust head-shoulder detection by PCA-based multilevel HOG-LBP detector for people counting. Proceedings of the International Conference on Pattern Recognition.

[10] Liu G., Yin Z., Jia Y., Xie Y. (2017). Passenger flow estimation based on convolutional neural network in public transportation system. Knowl. Base Syst..

[11] Gao C., Li P., Zhang Y., Liu J., Wang L. (2016). People counting based on head detection combining Adaboost and CNN in crowded surveillance environment. Neurocomputing.

[12] Wang Z., Cai G., Zheng C., Fang C. Bus-crowdedness estimation by shallow convolutional neural network. Proceedings of the International Conference on Sensor Networks and Signal Processing (SNSP).

[13] Chan A.B., Vasconcelos N. Bayesian Poisson regression for crowd counting. Proceedings of the IEEE International Conference on Computer Vision.

[14] Chen K., Loy C.C., Gong S., Xiang T. Feature mining for localised crowd counting. Proceedings of the British Machine Vision Conference (BMVC).

[15] Xu B., Qiu G. Crowd density estimation based on rich features and random projection forest. Proceedings of the IEEE Winter Conference on Applications of Computer Vision (WACV).

[16] Borstel M., Kandemir M., Schmidt P., Rao M., Rajamani K., Hamprecht F. Gaussian process density counting from weak supervision. Proceedings of the European Conference on Computer Vision (ECCV).

[17] Chan A.B., Liang Z.S.J., Vasconcelos N. Privacy preserving crowd monitoring: Counting people without people models or tracking. Proceedings of the IEEE Conference on Computer Vision and Pattern Recognition.

[18] Chan A.B., Vasconcelos N. (2012). Counting people with low-level features and Bayesian regression. IEEE Trans. Image Process..

[19] Idrees H., Saleemi I., Seibert C., Shah M. Multi-source multi-scale counting in extremely dense crowd images. Proceedings of the IEEE Conference on Computer Vision and Pattern Recognition.

[20] Lempitsky V., Zisserman A. Learning to count objects in images. Proceedings of the International Conference on Neural Information Processing Systems (NIPS), Hyatt Regency.

[21] Wang J., Wang L., Yang F. Counting crowd with fully convolutional networks. Proceedings of the International Conference on Multimedia and Image Processing (ICMIP).

[22] Zhang C., Li H., Wang X., Yang X. Cross-scene crowd counting via deep convolutional neural networks. Proceedings of the IEEE Conference on Computer Vision and Pattern Recognition (CVPR).

[23] Sindagi V.A., Patel V.M. (2017). CNN-based cascaded multi-task learning of high-level prior and density estimation for crowd counting. arXiv.

[24] Sindagi V.A., Patel V.M. (2017). Generating high-quality crowd density maps using contextual pyramid CNNs. arXiv.

[25] Zhang L., Shi M., Chen Q. (2017). Crowd counting via scale-adaptive convolutional neural network. arXiv.

[26] Zhang Y., Zhou D., Chen S., Gao S., Ma Y. Single-image crowd counting via multi-column convolutional neural network. Proceedings of the IEEE Conference on Computer Vision and Pattern Recognition (CVPR).

[27] Weng W.T., Lin D.T. Crowd density estimation based on a modified multicolumn convolutional neural network. Proceedings of the International Joint Conference on Neural Networks (IJCNN).

[28] Huang S., Li X., Zhang Z., Wu F., Gao S., Ji R., Han J. (2018). Body structure aware deep crowd counting. IEEE Trans. Image Process..

[29] Sang J., Wu W., Luo H., Xiang H., Zhang Q., Hu H., Xia X. (2019). Improved crowd counting method based on scale-adaptive convolutional neural network. IEEE Access.

[30] Yang B., Cao J., Wang N., Zhang Y., Zou L. (2018). Counting challenging crowds robustly using a multi-column multi-task convolutional neural network. Signal Process. Image Commun..

[31] Redmon J., Farhadi A. (2018). Yolov3: An incremental improvement. arXiv.

[32] Olmschenk G., Tang H., Zhu Z. (2019). Improving dense crowd counting convolutional neural networks using inverse k-nearest neighbor maps and multiscale upsampling. arXiv.

[33] Masci J., Meier U., Ciresan D., SchmidHuber J. Stacked convolutional auto-encoders for hierarchical feature extraction. Proceedings of the Artificial Neural Networks and Machine Learning (ICANN).

[34] Li Y., Zhang X., Chen D. (2018). CSRNet: Dilated convolutional neural networks for understanding the highly congested scenes. arXiv.

